# Effects of SGLT2 inhibitors on stroke and its subtypes in patients with type 2 diabetes: a systematic review and meta-analysis

**DOI:** 10.1038/s41598-021-94945-4

**Published:** 2021-07-28

**Authors:** Wen-Hsuan Tsai, Shih-Ming Chuang, Sung-Chen Liu, Chun-Chuan Lee, Ming-Nan Chien, Ching-Hsiang Leung, Shu-Jung Liu, Hong-Mou Shih

**Affiliations:** 1 Division of Endocrinology and Metabolism, Department of Internal Medicine, Mackay Memorial Hospital, No. 92, Sec. 2, Zhongshan N. Rd., Taipei, 10449 Taiwan; 2 Mackay Junior College of Medicine, Nursing, and Management, Taipei, Taiwan; 3 Department of Medicine, Mackay Medical Collage, New Taipei City, Taiwan; 4 Medical Library, MacKay Memorial Hospital, Tamsui Branch, New Taipei City, Taiwan; 5 Division of Nephrology, Department of Internal Medicine, Mackay Memorial Hospital, Taipei, Taiwan; 6 Graduate Institute of Physiology, College of Medicine, National Taiwan University, Taipei, Taiwan

**Keywords:** Type 2 diabetes, Stroke

## Abstract

Sodium-glucose cotransporter 2 (SGLT2) inhibitors have shown impressive effects in reducing major vascular events in several randomized controlled trials (RCTs). The purpose of this study was to perform a meta-analysis to evaluate the effect of SGLT2 inhibitors on the risk of stroke and its subtypes. All data from prospective RCTs up to 20 October 2020 involving SGLT2 inhibitors that reported stroke events as the primary endpoint or safety in subjects with type 2 diabetes were subjected to meta-analysis. Five eligible RCTs (EMPA-REG, CANVAS, DECLARE-TIMI 58, CREDENCE and VERTIS CV) involving 46,969 participants were included. Pooled analysis of the RCTs showed no significant effect of SGLT2 inhibitors on total stroke [risk ratio (RR) = 0.95; 95% confidence interval (CI) 0.79–1.13, P = 0.585]. Subgroup analysis indicated that SGLT2 inhibitors had no significant effect against fatal stroke, non-fatal stroke, ischemic stroke or transient ischemic attack. When only hemorrhagic stroke was included, SGLT2 inhibitors were associated with a significant 50% reduction compared with placebo (RR = 0.49, 95% CI 0.30–0.82, P = 0.007). This meta-analysis shows that SGLT2 inhibitors have a neutral effect on the risk of stroke and its subtypes but a potential protective effect against hemorrhagic stroke.

## Introduction

Type 2 diabetes mellitus (T2DM) is a highly prevalent disease associated with an increased risk of macrovascular complications (such as acute myocardial infarction and stroke), accounting for 80% of deaths in diabetic individuals^1^. T2DM patients with incident stroke have a higher risk of mortality than those with diabetes alone^2^. Sodium-glucose cotransporter 2 (SGLT2) inhibitors are a novel class of oral hypoglycemic agents for the treatment of T2DM that have a unique antidiabetic mechanism that inhibits the proximal renal tubule’s reabsorption of glucose and sodium, thus reducing the level of blood glucose^3^. With the results of cardiovascular outcome trials for SGLT2 inhibitor and glucagon-like peptide-1 receptor agonists (GLP-1RA), there is considerable evidence that these treatment modalities can lower the risk of major cardiovascular disease (CVD) outcomes, including non-fatal stroke^4^. Although previous relevant meta-analyses showed that SGLT2 inhibitors have a neutral effect on the risk of stroke^5–8^, these randomized controlled trials (RCTs) focused on statistical analysis of non-fatal strokes from cardiovascular events without distinguishing between the different stroke subtypes, such as fatal, transient ischemic attack (TIA), ischemic and hemorrhagic.

Risk for the development of stroke may vary according to the patients’ characteristics, risk factors and etiologies of stroke^9^. From some studies, we found that the effect of SGLT2 inhibitors on the risk of stroke differed according to the stroke subtype (ischemic or hemorrhagic)^10,11^. Very few meta-analyses or systematic reviews have investigated the effects of SGLT2 inhibitors on the risk of stroke in patients with T2DM, therefore a meta-analysis of all relevant published literature from RCTs was performed to qualitatively and quantitatively investigate the effects of SGLT2 inhibitors on the risk of stroke in diabetic individuals.

## Materials and methods

### Search strategy and selection criteria

We conduct this study in accordance with the recommendations of the Cochrane Collaboration and PRISMA (Preferred Reporting Items for Systematic Reviews and Meta-Analysis) guidelines and registered on PROSPERO (CRD42021224672).

### Eligibility criteria

We searched PubMed, EMBASE and Cochrane Central Register of Controlled Trials databases for RCTs published until 20 October 2020, with no language restrictions. The key research terms used were “SGLT2”, “canagliflozin”, “dapagliflozin”, “empagliflozin”, “ertugliflozin”, “stroke”, “ischemic stroke”, “hemorrhagic stroke”, “transient ischemic attack”, “fatal stroke”, “non-fatal stroke”, “cerebrovascular disease”, “cerebral ischemia”, “myocardial infarction”, “coronary heart disease”, “cardiovascular disease” OR “coronary artery disease”. The other search terms are listed in Supplementary Table S1.

Criteria for inclusion of a study derived from all previously published meta-analysis in this area were: (1) RCTs design; (2) comparison of SGLT2 inhibitors with control (eg, placebo or other glucose-lowering agents); and (3) Studies with at least 1000 patients above 18 years of age. (4) Studies with at least 18 months follow up (5) studies that reported an outcome of cerebrovascular disease (disease was defined as any fatal or non-fatal ischemic stroke, hemorrhagic stroke, or transient ischemic attack) or stroke events as the primary endpoint or safety in subjects with type 2 diabetes. Other retrospective database and non-randomized studies were excluded.

Two authors (Tsai and Chuang) independently reviewed and screened each title and abstract from related publications of eligible studies and conference abstracts since 20 September 2017. The full texts were reviewed if they corresponded to our outcomes. Two reviewers (Liu and Lee) collected and analyzed information on outcomes and baseline characteristics. All selected studies were enrolled in accordance with the eligibility criteria. When consensus could not be obtained between the two screening authors, a third author (Chien) was independently consulted to resolve any issues. All duplicate data, letters to the editor, case series and studies on type 1 diabetes were excluded from our research.

### Data extraction and quality assessment

All data were extracted from the enrolled studies by two independent investigators according to a standard protocol. Data variables were recorded as follows: study name, Clinical Trial Registration Number (NCT ID), number of participants, age, gender, body mass index (BMI), follow-up duration, CVD (%), heart failure (%), chronic kidney disease (CKD, %), mean hemoglobin A1c test (HbA1c) and outcomes of interest for each group. We assessed the quality of individual studies by the Cochrane risk-of-bias algorithm (www.cochrane.org/training/cochrane-handbook).

### Data synthesis and analysis

We present a descriptive analysis of each individual trial (Table 1). The primary efficacy outcome analyzed was stroke events (non-fatal, fatal, ischemic, hemorrhagic, TIA). The risk ratio (RR) and 95% confidence interval (CI) of stroke outcomes from individual studies were analyzed using a random-effect model. Weighted pooled treatment effects were also calculated. Heterogeneity across studies was assessed with the I^2^ statistic and the variability of studies was estimated as heterogeneous if the χ^2^ test was significant (*P* < 0.05) or the I^2^ statistic was > 50%. We assessed possible publication/disclosure bias using funnel plots and Begg’s test. Comprehensive Meta-Analysis (CMA) V3 software (Borenstein M et al. Comprehensive Meta-analysis Version 3, Biostat, Englewood NJ, 2014) was use for calculating meta-analysis.

**Table 1:** Baseline characteristics of trials included in the systematic review and meta-analysis.

Studies	EMPA-REG	CANVAS	DECLARE-TIM 58	CREDENCE	VERTIS CV
NCT ID	NCT01131676	NCT01032629/NCT01989754	NCT01730534	NCT02065791	NCT01986881
Study participants	7020	10,142	17,160	4401	8246
Intervention	Empagliflozin	Canagliflozin	Dapagliflozin	Canagliflozin	Ertugliflozin
Control	Placebo	Placebo	Placebo	Placebo	Placebo
Mean age(years)	63.1 ± 8.6	63.3 ± 8.3	63.9 ± 6.8	63.0 ± 9.2	64.4 ± 8.1
Male (%)	71.5	64.2	62.6	66.1	70
BMI	30.6 ± 5.2	32.0 ± 5.9	32.1 ± 6.0	31.3 ± 6.2	NA
Follow-up years	3.1	2.4	4.2	2.6	3
DM duration (years)	NA	13.5 ± 7.8	11.8 ± 7.8	15.8 ± 8.6	13.0 ± 8.3
CVD (%)	100	65.6	40.6	50.4	100
HF (%)	10.1	14.4	10	14.8	23.7
CKD (%)	25.9	20.1	7.4	59.8	21.9
Mean HbA1C (%)	8.1 ± 0.8	8.2 ± 0.9	8.3 ± 1.2	8.3 ± 1.3	8.2 ± 1.0

*BMI* body mass index, *CVD* cardiovascular disease, *HF* heart failure, *CKD* chronic kidney disease, *Hba1c* hemoglobin *A1c*, *EMPA-REG OUTCOME Empagliflozin*, cardiovascular outcomes, and mortality in type 2 diabetes, *CANVAS* Canagliflozin and cardiovascular and renal events in type 2 diabetes, *DECLARE-TIMI 58* dapagliflozin and cardiovascular outcomes in type 2 diabetes, *CREDENCE* canagliflozin and renal outcomes in type 2 diabetes and nephropathy, *VERTIS CV* Cardiovascular outcomes with Ertugliflozin in type 2 diabetes.

## Results

**Figure 1:**
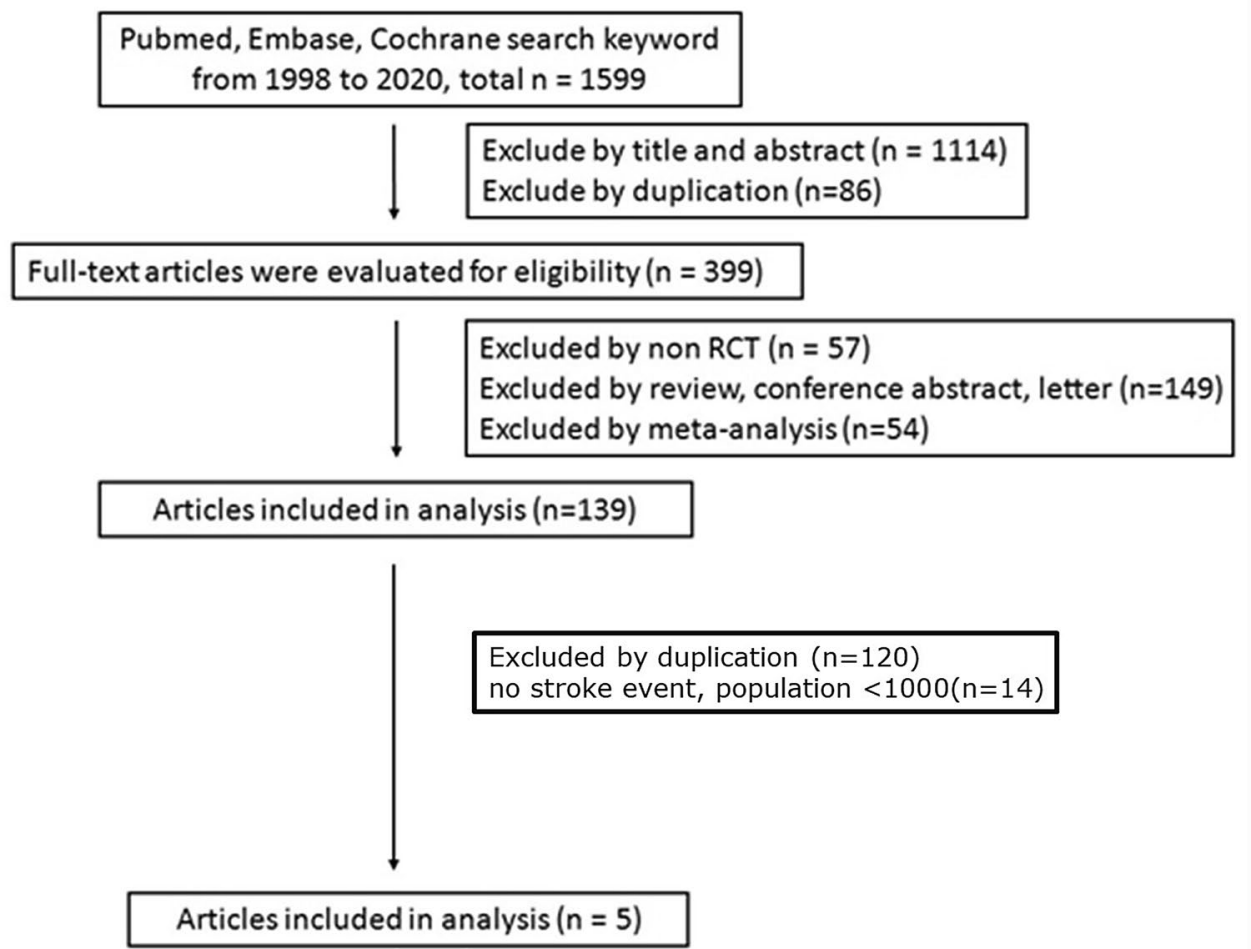
Flow chart showing selection of studies for meta-analysis.

A flowchart of the literature search is shown in Fig. 1. Initial implementation of the search strategy yielded 1599 potentially relevant citations. The literature review identified 139 articles for further assessment, of which 95 were excluded due to duplication, 25 due to clinical trial design and 14 because of absence of stroke events. According to the predetermined criteria, a total of five studies involving 46,969 T2DM patients published between 2015 and 2020 were eligible for inclusion in the meta-analysis.

The literature search identified a total of five trials that met our criteria, with all analyses mainly conducted on the following trials: EMPA-REG OUTCOME (Empagliflozin, Cardiovascular Outcomes, and Mortality in Type 2 Diabetes)^5^, CANVAS (Canagliflozin and Cardiovascular and Renal Events in Type 2 Diabetes)^6^, DECLARE-TIMI 58 (Dapagliflozin and Cardiovascular Outcomes in Type 2 Diabetes)^7^, CREDENCE (Canagliflozin and Renal Outcomes in Type 2 Diabetes and Nephropathy)^8^, and VERTIS CV (Cardiovascular Outcomes with Ertugliflozin in Type 2 Diabetes)^12^.

The baseline characteristics of the patients in the selected RCTs are shown in Table 1. The number of study participants ranged from 4401 to 17,160 and the duration of intervention was 2.4–4.2 years. Among the studies enrolled for meta-analysis, DECLARE-TIMI 58 had at least 40% of participants with established CVD, EMPA-REG and VERTIS CV had almost 100% with CVD and another two trials (CANVAS, CREDENCE) had 65% and 50% with CVD, respectively. All participants in this study had a mean estimated glomerular filtration rate (eGFR) in the range 56–85 mL/min/1.73 m^2^. Only the CREDENCE trial had a greater number of patients with CKD (around 60%) while the other four trials had 7.4–25.9% of patients with CKD. Five studies were analyzed to assess the effects of SGLT2 inhibitors on the risk of stroke in T2DM patients. Analyzed data were abstracted from whole trials that enrolled non-fatal or fatal stroke patients (all five trials) and additionally reported on stroke patients from other subgroups, including hemorrhagic (three trials), ischemia (five trials) and TIA (three trials). SGLT2 inhibitors were compared with placebo in all five studies.

**Figure 2:**
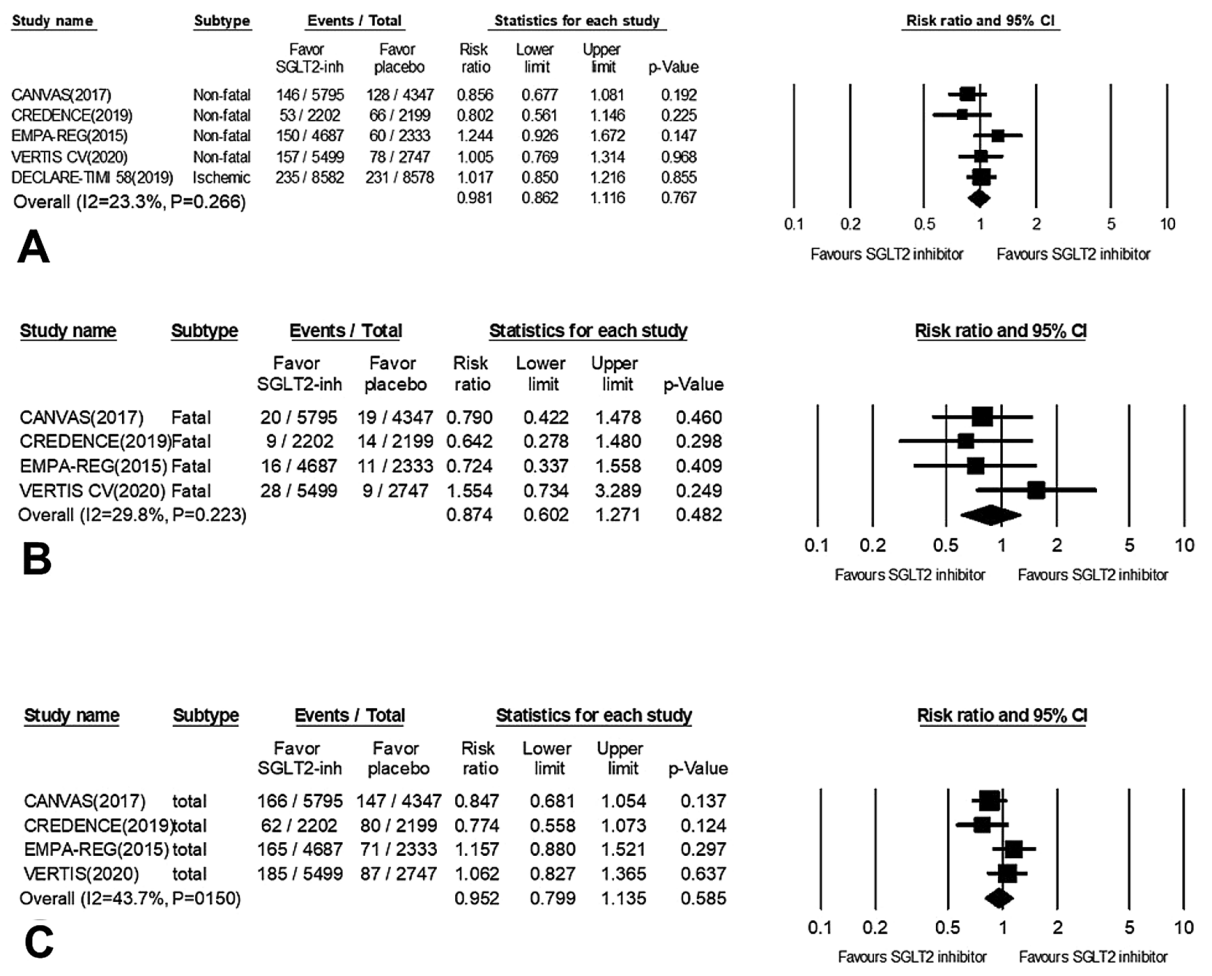
Forest plots for meta-analysis of the effects of SGLT2 inhibitors on stroke outcomes: (**A**) non-fatal stroke; (**B**) fatal stroke and (**C**) total stroke. Summary effects for all drugs were obtained from a random-effect model.

**Figure 3:**
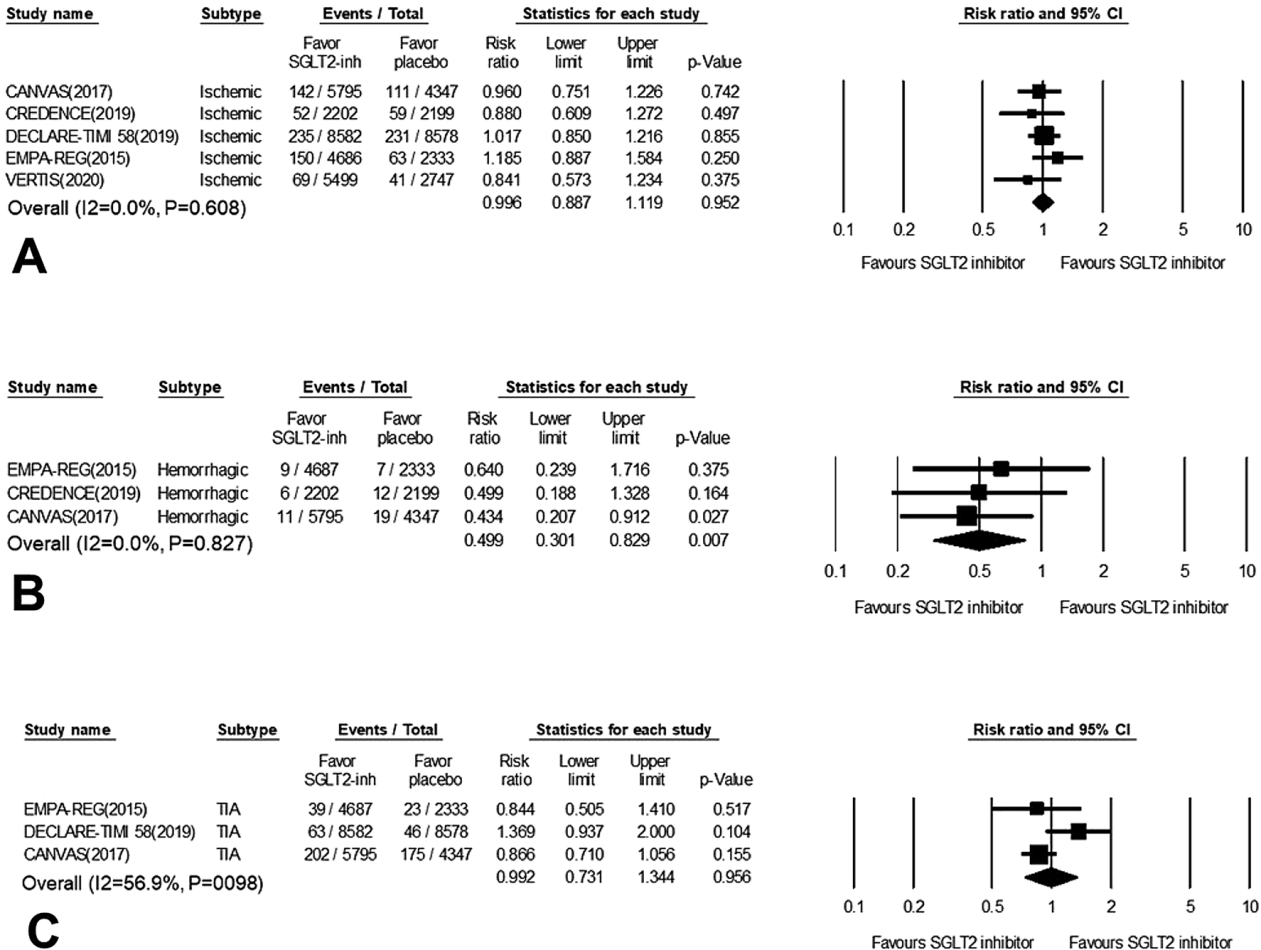
Forest plots for meta-analysis of the effects of SGLT2 inhibitors on stroke subtype: (**A**) ischemic stroke; (**B**) hemorrhagic stroke and (**C**) transient ischemic attack (TIA). Summary of effects for all drugs were obtained from a random-effect model.

The five studies collected for meta-analysis of the effect of SGLT2 inhibitors on stroke are listed in Figs. 2 and 3. Pooled analysis reported no significant effect on total stroke outcomes from treatment with SGLT2 inhibitors versus placebo (RR = 0.95, 95% CI 0.79–1.13, *P* = 0.585; Fig. 2], with mild effect heterogeneity across all studies (I^2^ = 43.7%, *P* for interaction = 0.150). Similarly, with respect to other stroke subtype outcomes, subgroup analysis indicated that SGLT2 inhibition had no significant effect on either fatal (RR = 0.87, 95% CI 0.60–1.27, *P* = 0.482; Fig. 2B) or non-fatal stroke (RR = 0.98, 95% CI 0.86–1.11, *P* = 0.767; Fig. 2A).

For ischemic stroke, SGLT2 had a neutral effect (RR = 0.99, 95% CI 0.88–1.11, *P* = 0.952) without any significant heterogeneity (I^2^ = 0%, *P* for interaction = 0.608; Fig. 3A). When only hemorrhagic stroke was included (this applied for the EMPA-RAG, CANVAS and CREDENCE trials), active treatment with SGLT2 inhibitors was associated with a significant 50% reduction (RR = 0.49, 95% CI 0.30–0.82, *P* = 0.007), with no evidence of heterogeneity between studies (I^2^ = 0%, *P* for interaction = 0.827; Fig. 3B). On the other hand, analysis of the three RCTs (EMPA-REG, DECLARE-TIMI 58 and CANVAS) showed no differences in the risk of TIA compared with a placebo (RR = 0.99, 95% CI 0.73–1.34, *P* = 0.956; Fig. 3C). There was also no significant trial heterogeneity (I^2^ = 56.9%, *P* for interaction = 0.098).

## Discussion

Although SGLT2 inhibitors have shown an impressive positive effect on reducing composite major adverse cardiovascular events^13^, previous meta-analyses demonstrated that the effect of treatment with SGLT2 inhibitors was neutral on stroke outcome^14^. New meta-analysis research was necessary because several novel RCTs of SGLT2 inhibitors on CVD outcomes were published recently and few studies have investigated the effect of SGLT2 on different stroke subtype outcomes. Therefore, we conducted pooled analyses to compare the effect of SGLT2 inhibitors on stroke outcome and the individual outcomes for different stroke subtypes. This meta-analysis of the data derived from five RCTs involving 46,969 participants showed that treatment with SGLT2 inhibitors did not increase the risk of total stroke events and there was a neutral effect among different stroke subtypes apart from hemorrhagic stroke. Furthermore, all data derived from large RCTs showed that treatment with SGLT2 inhibitors had a safe and neutral effect on the risk of stroke, with a potential beneficial effect on hemorrhagic stroke over placebo. These results provided sufficient evidence for safety issues but would benefit from further research in the future.

There are meta-analyses of glucose-lowering drugs that support the evidence of potential stroke-protective effects from treatment with pioglitazone and GLP-1R^15,16^, whereas dipeptidyl peptidase-4 (DPP-4) inhibitors have a neutral effect against stroke^17^. Earlier studies were concerned that SGLT2 inhibitors may increase the risk of ischemic stroke due to volume depletion, orthostatic hypotension and dehydration caused by a weak loop diuretic effect from the SGLT2 inhibitors^18^. Previous meta-analyses conducted by Guo et al. proved that SGLT2 inhibitors did not increase stroke incidence but they did not classify the stroke subtypes^14^. Based on the eligibility criteria in our study, data from RCTs involving SGLT2 inhibitors that reported total stroke events or its subtypes (including fatal, non-fatal, ischemic, hemorrhagic or TIA) as primary endpoints or safety were selected for further meta-analysis. These results provided a more comprehensive evaluation of the effect of SGLT2 inhibitors on the risk of stroke and its subtypes. The available data in our research were acquired from large prospective RCTs that have a longer intervention duration, more rigorous design and more stroke events. Additionally, in order to reduce the heterogeneity from different studies, our control groups were treated with placebo instead of specific classes of drugs. The meta-analysis conducted by Zhou et al. did not include the VERTIS CV trial for analysis, unlike our study. Both studies revealed consistent results that SGLT2 inhibitors have a neutral effect on the risk of stroke as compared with placebo^19^.

One cohort study showed that canagliflozin did not significantly decrease the risk of stroke compared with DPP-4 inhibitor, sulfonylurea or GLP-1R agonist^20^. In addition, the CREDENCE trial published in 2019 revealed that canagliflozin demonstrated no significant differences in reduction of fatal stroke, non-fatal stroke, ischemic stroke or TIA but there was a significant reduction of hemorrhagic stroke compared with placebo^8^. Possible mechanisms to explain this unsatisfactory study result may be different baseline characteristics, insufficient follow-up duration, a history of cerebrovascular events, dehydration, hemoconcentration or hyperglycemia. Kenichiro Sato et al. performed a disproportionality analysis using the Japanese Adverse Drug Event Report database and advocated that SGLT2 inhibitors are associated with an increased risk of ischemic stroke, including thrombosis, lacunar infarction and embolism, but there was no significantly higher risk for hemorrhagic stroke^10^. The discrepancy in the effect of SGLT2 inhibitors on each stroke subtype may have masked the overall incidence of stroke, resulting in the non-significant stroke risk conclusions in the previous RCTs. In our study, although SGLT2 inhibitors showed no significant benefit on total stroke; the CANVAS program and the CREDENCE trial showed that there were heterogeneity effects on renal function and a significant risk reduction of stroke in, especially for subjects with lower eGFR. Most findings about the effects of SGLT2 inhibitors on stroke were from participants with normal renal function. Due to lack of evidence to verify the above hypothesis, further research is warranted to explore the impact of SGLT2 inhibitors on stroke or stroke subtypes in diabetes patients with significantly impaired renal function.

Poorly controlled hyperglycemia reduces cerebral blood flow and oxygenation of tissues, and increases intracranial pressure, cerebral edema and neuronal death, which are more severe in patients with diabetes^21,22^. However, intensive diabetes management cannot reduce the risk of stroke in patients with T2DM^23^. Beyond glucose lowering, novel hypoglycemic agents have more pleiotropic properties for reducing vascular events over traditional hypoglycemic drugs such as GLP-1R agonists and SGLT2 inhibitors^4^. SGLT2 inhibitors decrease cardiovascular events via multiple possible mechanisms, such as reducing glucose, blood pressure, weight, arteriosclerosis and albuminuria. In animal models, SGLT2 inhibitors have protective effects on CVD by suppressing inflammation and oxidative stress, reversing pro-inflammatory status and glucotoxicity and mediating AGE/RAGE (Advanced glycation end products/Receptor for advanced glycation end products) signaling in diabetic rodents^24^. Empagliflozin showed improvements in inflammation and insulin resistance, which may cause mitigation of atherosclerosis^25^. In rodent studies, β-hydroxybutyrate, a ketone body that is increased after SGLT2 inhibitor treatment, could reduce brain infarct size by activating neuro-protective macrophages and also shift glucose metabolism towards reducing oxidative stress^26^. Increase of the hematocrit may increase delivery of oxygen to tissues, which should reduce neural damage^27^. Besides, atrial fibrillation plays an important role in the development of stroke risk, and SGLT2 inhibitors also showed some promising effect on the reduction of atrial fibrillation^28^.

In our meta-analysis, we conducted pooled analysis with the EMPA-REG, CANVAS and CREDENCE trials and found that SGLT2 inhibitors caused a significant 50% reduction in hemorrhagic stroke compared with placebo. Although there was no beneficial effect on ischemic, TIA, non-fatal or fatal stroke, potential protection of SGLT2 inhibitors against hemorrhagic stroke was shown in pooled analysis of the different stroke subtypes, despite the small number of events. The results for hemorrhagic stroke may be attributed to blood pressure reduction, one of the mechanisms of stroke prevention arising from canagliflozin use. Compared with ischemic stroke, higher blood pressure produces a higher risk of hemorrhagic stroke^29^. As we know, blood pressure lowering is associated with a decreased risk of stroke, especially for the hemorrhagic subtype^30^. This explains why canagliflozin has a beneficial effect on stroke based upon blood pressure reduction. SGLT2 inhibitors decrease blood pressure by multiple related mechanisms including diuresis, glucouresis, natriuresis, dehydration and weight loss^31^. They also increase cholesterol levels, although this has little effect; but may be helpful in preventing hemorrhagic stroke. Some of the benefits of SGLT2 inhibitors such as weight reduction and glucose lowering may improve atherosclerosis, which is also related to long-term reduction of the risk of stroke^32^. The possible beneficial renal and cardiovascular outcomes from SGLT2 inhibitors were attributed to elevation of the hematocrit^33^. Although some cohort studies have shown that an increased hematocrit is associated with a reduced risk of hemorrhagic stroke^34,35^, current evidence is still uncertain whether the increased hematocrit caused by SGLT2 inhibitors can lower the risk of hemorrhagic stroke. Regarding continuation or discontinuation of treatment, the hazard ratio of SGLT2 inhibitors on stroke was steady^36^, and it is currently difficult to prove that patient withdrawal from randomized treatment has any adverse effect on the risk of stroke.

Although we conducted a comprehensive systematic review of RCTs for SGLT2 inhibitors, there were some limitations. Firstly, none of the included trials were designed specifically to assess the cerebrovascular outcomes of SGLT2 inhibitors, however, all trials were intended to evaluate the safety of SGLT2 inhibitors. Furthermore, the included trials covered a wide range of clinical characteristics, such as age, disease duration and follow-up duration, which inevitably led to heterogeneity. Secondly, only five large RCTs met the inclusion criteria for our meta-analysis and they have a relatively long-term follow-up duration of at least 2 years. We excluded small prospective trials due to small sample sizes, short-term follow-up and few stroke events. Thirdly, despite a significant reduction risk of hemorrhagic stroke, whether SGLT2 inhibitor use is beneficial for the prevention of hemorrhagic stroke requires further investigation due to the small numbers of events recorded in our analysis. These limitations may impair the power of our study. However, with a comprehensive literature search covering three databases, most of the eligible studies selected by two different investigators according to strict inclusion criteria were of moderate to high quality. Therefore, we believe that it is reasonable to draw conclusions from this meta-analysis.

## Conclusions

SGLT2 inhibitors have a neutral effect on overall cerebrovascular events but outcomes for stroke following the use of SGLT2 inhibitors differ depending on the stroke subtypes, with a potential benefit for prevention of hemorrhagic stroke. This suggests the need for further prospective studies to compare the effects of SGLT2 inhibitors on different stroke subtypes.

## Supplementary Information

Supplementary Information.
