# Supplementary material for: Effects of SGLT2 inhibitors on stroke and its subtypes in patients with type 2 diabetes: a systematic review and meta-analysis
Source: Sci Rep. 2021 Jul 28;11:15364. doi: 10.1038/s41598-021-94945-4 (PMC8319393; doi:10.1038/s41598-021-94945-4)

**Supplementary Appendix**

**Table S1.** Search strategy.

**Table S2**. Risk-of-Bias Assessment of Included Trials

**Table S3.** Different subtype of stroke events from five large SGLT2 inhibitor trials were record included in the systematic review and meta-analysis

**Figure S4.** Funnel plot of standard error by log relative risk for SGLT2 inhibitors and control groups to detect publication bias.

**Table S1.** Search Strategy

| **Data source** | **Search terms** |
| --- | --- |
| **PubMed** | 1. “sodium glucose cotransporter”[Text Word] OR “sodium-glucose-cotransporter-2 inhibitors”[Text Word] OR “sglt-2”[Text Word] OR “sglt2”[Text Word] OR “canagliflozin”[Text Word] OR “dapagliflozin”[Text Word] OR “empagliflozin”[Text Word] OR “ ertugliflozin”[Text Word] OR “Ipragliflozin”[Text Word] OR “ luseogliflozin”[Text Word] OR “ remogliflozin”[Text Word] OR “sotagliflozin”[Text Word] 2. “stroke”[Text Word] OR “ischemic stroke”[Text Word] OR “hemorrhagic stroke”[Mesh Terms] OR “ transient ischemic attack”[Mesh Terms] OR “fatal stroke”[Mesh Terms] OR “non-fatal stroke”[Mesh Terms] OR "cardiovascular disease”[Mesh Terms] OR “coronary artery disease”[Mesh Terms] OR “coronary heart disease”[Text Word] OR “myocardial infarction”[Mesh Terms] OR “macrovascular disease”[Text Word] OR “cerebrovascular disease”[Text Word] OR “cerebral ischemia”[Text Word] OR “mortality”[Mesh Terms] OR “safety”[Mesh Terms] 3. #1 AND #2 |
| **CENTRAL** | Title,Abstract,Keywords ((‘Sodium glucose cotransporter’ OR ‘SGLT 2’ OR ‘SGLT2’OR canagliflozin OR dapagliflozin OR empagliflozin OR ertugliflozin OR Ipragliflozin OR luseogliflozin OR remogliflozin OR sotagliflozin) AND (stroke OR ischemic stroke’ OR ‘hemorrhagic stroke’ OR ‘ fatal stroke’ or ‘non-fatal stroke’ OR ‘transient ischemic attack’ OR ‘cerebrovascular disease’ OR ‘cerebral ischemia’ OR ‘myocardial infarction’ OR ‘coronary heart disease’ OR ‘cardiovascular disease’ OR ‘coronary artery disease’ OR ‘macrovascular disease’)) AND (‘trials’) |
| **Embase** | Title-Abstract-Author Keywords ((‘Sodium glucose cotransporter’ OR ‘SGLT 2’ OR ‘SGLT2’ OR ‘canagliflozin’ OR ‘dapagliflozin’ OR ‘empagliflozin’ OR ‘ertugliflozin’ OR ‘Ipragliflozin’ OR ‘luseogliflozin’ OR ‘remogliflozin’ OR ‘sotagliflozin’) AND (stroke OR ischemic stroke’ OR ‘hemorrhagic stroke’ OR ‘ fatal stroke’ or ‘non-fatal stroke’ OR ‘transient ischemic attack’ OR ‘cerebrovascular disease’ OR ‘cerebral ischemia’ OR ‘myocardial infarction’ OR ‘coronary heart disease’ OR ‘cardiovascular disease’ OR ‘coronary artery disease’ OR ‘macrovascular disease’)) AND TITLE-ABSTRACT-INDEX TERM (RCT* OR random*) |
| **Web of Science** | TS=((‘Sodium glucose cotransporter’ OR ‘SGLT 2’ OR ‘SGLT2’ OR canagliflozin OR dapagliflozin OR empagliflozin OR ertugliflozin OR Ipragliflozin OR luseogliflozin OR remogliflozin OR sotagliflozin) AND (stroke OR ischemic stroke’ OR ‘hemorrhagic stroke’ OR ‘ fatal stroke’ or ‘non-fatal stroke’ OR ‘transient ischemic attack’ OR ‘cerebrovascular disease’ OR ‘cerebral ischemia’ OR ‘myocardial infarction’ OR ‘coronary heart disease’ OR ‘cardiovascular disease’ OR ‘coronary artery disease’ OR ‘macrovascular disease’)) AND (‘clinical trials’) |
| **Scopus** | TITLE-ABS-KEY ((“Sodium glucose cotransporter” OR “SGLT 2” OR “SGLT2”OR canagliflozin OR dapagliflozin OR empagliflozin OR ertugliflozin OR Ipragliflozin OR luseogliflozin OR remogliflozin OR sotagliflozin) AND (stroke OR ischemic stroke’ OR ‘hemorrhagic stroke’ OR ‘ fatal stroke’ or ‘non-fatal stroke’ OR ‘transient ischemic attack’ OR ‘cerebrovascular disease’ OR ‘cerebral ischemia’ OR ‘myocardial infarction’ OR ‘coronary heart disease’ OR ‘cardiovascular disease’ OR ‘coronary artery disease’ OR ‘macrovascular disease)) AND TITLE-ABS-KEY ( rct*  OR "randomized controlled trial" ) AND  ( LIMIT-TO ( DOCTYPE ,  "ar" ) )  AND  ( LIMIT-TO ( EXACTKEYWORD ,  "Human" ) |
| **ClinicalTrials.gov** | Tofogliflozin OR Empagliflozin OR dapagliflozin OR Canagliflozin OR Sotagliflozin OR luseogliflozin OR Ipragliflozin OR ertugliflozin |
|  |  |

**Table S2**. Risk-of-Bias Assessment of Included Trials

| **Domain** | EMPA-REG OUTCOME | CANVAS | DECLARE-TIMI 58 | CREDENCE | VERTIS CV |
| --- | --- | --- | --- | --- | --- |
| Random sequence generation (selection bias) | Low | Low | Low | Low | Low |
| Allocation concealment (selection bias) | Low | Low | Low | Low | Low |
| Blinding of participants and personnel (performance bias) | Low | Low | Low | Low | Low |
| Blinding of outcome assessment (detection bias) | Low | Low | Low | Low | Low |
| Incomplete outcome data (attrition bias) | Low | Low | Low | Low | Low |
| Selective reporting (reporting bias) | Low | Low | Low | Low | Low |
| Other potential bias | Low | Low | Low | Low | Low |

| **Table S2** Different subtype of stroke events from five large SGLT2 inhibitor trials were record included in the systematic review and meta-analysis | | | | | | | | | |
| --- | --- | --- | --- | --- | --- | --- | --- | --- | --- |
| Study | NCT ID | Participants(N) | Intervention | control | subgroup | Treated events | Treated total N | Control events | Control total N |
| EMPA REG OUTCOME | NCT01131676 | 7020 | EMPA | placebo | nonfatal | 150 | 4687 | 60 | 2333 |
|  |  |  |  |  | fatal | 16 | 4687 | 11 | 2333 |
|  |  |  |  |  | TIA | 39 | 4687 | 23 | 2333 |
|  |  |  |  |  | Ischemic | 150 | 4687 | 63 | 2333 |
|  |  |  |  |  | Hemorrhagic | 9 | 4687 | 7 | 2333 |
| CANVAS | NCT01032629 | 10142 | CANA | placebo | Nonfatal | 146 | 5795 | 128 | 4347 |
|  |  |  |  |  | Fatal | 20 | 5795 | 19 | 4347 |
|  |  |  |  |  | Ischemic | 142 | 5795 | 111 | 4347 |
|  |  |  |  |  | Hemorrhagic | 11 | 5795 | 19 | 4347 |
|  |  |  |  |  | TIA | 202 | 5795 | 175 | 4347 |
| DECLARE-TIMI58 | NCT01730534 | 17160 | DAPA | placebo | ischemic | 235 | 8582 | 231 | 8578 |
|  |  |  |  |  | TIA | 63 | 8582 | 46 | 8578 |
| CREDENCE | NCT02065791 | 4401 | CANA | placebo | Nonfatal | 53 | 2202 | 66 | 2199 |
|  |  |  |  |  | Fatal | 10 | 2202 | 14 | 2199 |
|  |  |  |  |  | Ischemic | 52 | 2202 | 59 | 2199 |
|  |  |  |  |  | Hemorrhagic | 6 | 2202 | 12 | 2199 |
| VERTIS CV | NCT01986881 | 8246 | ERTU | placebo | Nonfatal | 157 | 5499 | 78 | 2747 |
|  |  |  |  |  | Fatal | 28 | 5499 | 9 | 2747 |
|  |  |  |  |  | Ischemic | 69 | 5493 | 41 | 2745 |

**Figure S3.** Funnel plot of standard error by log relative risk for SGLT2 inhibitors and control groups to detect publication bias.


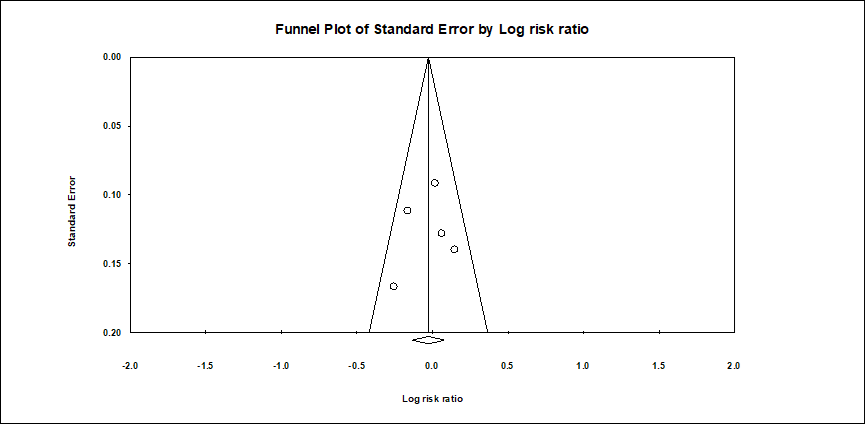

Supplement: Supplementary file 1 — Supplementary Information. [file 41598_2021_94945_MOESM1_ESM.docx]
